# The increased risk of cyberbullying and its negative impact on mental health among sexual minority college students compared to their peers

**DOI:** 10.1186/s13034-025-00895-4

**Published:** 2025-04-02

**Authors:** Man Luo, Zhizhou Duan, Wenqun Luo, Zhiping Niu

**Affiliations:** 1 Department of Public Health, School of medicine, Yangtze University, Jingzhou, Hubei,, China; 2 Preventive Health Service, Jiangxi Provincial People’s Hospital, The First Affiliated Hospital of Nanchang Medical College, Nanchang, Jiangxi China; 3 Gynecology Department, Jiangxi Provincial People’s Hospital, The First Affiliated Hospital of Nanchang Medical College, Nanchang, Jiangxi China; 4 Department of Environmental Health, School of Public Health, Fudan University, 130 Dong’an Road, Shanghai, 200032 China

**Keywords:** Sexual minorities, Cyberbullying, Mental health, Mediation

## Abstract

**Backgrounds:**

This study explores the relationship between cyberbullying, mental health, and sexual minority groups, focusing on the specific impact of cyberbullying on these individuals’ mental wellbeing. Previous research has indicated that sexual minorities face higher levels of cyberbullying on social media. Therefore, this study aims to gain deeper insights into the mental health consequences and potential mitigating factors for this vulnerable population.

**Methods:**

We employed a questionnaire survey method and convenience sampling to collect data. Participants’ psychosocial traits were assessed using established scales measuring stress, anxiety, depression, cyberbullying, loneliness, and sleep quality. Data analysis included Propensity Score Matching (PSM) and causal mediation analysis.

**Results:**

Of the participants, 204 identified as belonging to sexual minority groups, constituting 7.1% of the overall sample. The correlation results indicated that individuals in the sexual minority group experienced higher levels of cyberbullying (*r* = 0.276, *p* < 0.001). Cyberbullying was found to partially mediate the relationship between sexual minority status and several mental health issues, including depressive symptoms (0.32, 95% CI 0.19–0.53; *P* < 0.001), anxiety symptoms (0.52, 95% CI 0.30–0.88; *P* < 0.001), and loneliness (0.40, 95% CI 0.23–0.69; *P* < 0.001). Additionally, cyberbullying fully mediated the relationship between sexual minority status and both stress and sleep quality.

**Conclusion:**

These findings highlight that cyberbullying serves as a significant mediator in the mental health challenges faced by sexual minorities.

## Introduction

With the advancement of technology, the Internet has become an integral part of everyday life. However, this digital landscape also presents significant risks, one of which is cyberbullying [1]. Often referred to as online or electronic bullying, cyberbullying occurs through various digital platforms, including mobile phones, computers, and tablets [2]. It can manifest on social media sites, messaging apps, gaming platforms, and other spaces where individuals can engage with or share content. Social media is particularly notorious for facilitating cyberbullying, with platforms like Facebook, Instagram, Snapchat, TikTok, and Weibo (in China) being among the most common venues for such behavior [3]. For college students who are new to university life, the threat of prolonged cyberbullying is especially concerning. This demographic often grapples with the challenges of adjusting to unfamiliar environments, navigating shifts in social circles, and exploring their identities—factors that can heighten their vulnerability to online harassment.

Previous research has indicated that cyberbullying is prevalent among adolescents. A review revealed that approximately 10–15% of college students have encountered cyberbullying during their university years [4]. Furthermore, Huang et al. reported that the incidence of cyberbullying among Chinese college students can reach as high as 64.3% [5]. The adverse effects of cyberbullying on mental health are well-documented. For instance, Grigore et al. found a significant positive correlation between cyberbullying and anxiety levels among middle school students [6]. A study conducted in Bangladesh indicated that the prevalence of major depressive disorder among victims of cyberbullying was 9.1%, which is higher than that of non-victims [7]. Additionally, Jin et al. discovered that Chinese middle school students in Chongqing who experienced cyberbullying were more likely to attempt suicide [8].

Sexual minority students are particularly vulnerable in the online environment, making them more susceptible to cyberbullying [9]. This vulnerability is closely linked to the social identities they form, which are often shaped by societal constructs that carry prejudice, stereotypes, and discrimination. When bullying behavior rooted in social prejudice manifests on digital platforms, it becomes known as prejudice-based cyberbullying [10]. Numerous studies have demonstrated that LGBTQ + individuals face a significantly higher risk of experiencing bullying and harassment in online spaces compared to their heterosexual peers. This heightened exposure to cyberbullying is strongly associated with increased suicide risk, deteriorating mental health, and declining academic performance [11, 12]. Additionally, a systematic review of 27 empirical studies has found that the prevalence of cyberbullying among LGBTQ + adolescents ranges from 10.5 to 71.3%. Notably, LGBTQ + students report experiencing cyberbullying at higher rates than their heterosexual counterparts, underscoring the heightened vulnerability of sexual minority groups in the digital landscape [12].

Previous research has shown that sexual minority groups are more likely to experience higher levels of cyberbullying on social media. For instance, a 2015 survey by Cénat et al. found that bisexual youth may be particularly vulnerable to cyberbullying compared to their heterosexual peers [13]. Furthermore, numerous studies have demonstrated that both male and female sexual minority youth report significantly higher levels of cyberbullying than their heterosexual and cisgender counterparts (e.g., Schneider et al., 2015; Wensley & Campbell, 2012) [14, 15]. However, the specific impact of cyberbullying on the mental health of these individuals remains largely unexplored [12]. According to the Minority Stress Model [16], sexual minorities experience chronic and multifaceted social pressures as a result of their identity characteristics. Cyberbullying, as a specific manifestation of these social pressures in the online realm, not only inflicts direct psychological harm on sexual minorities but also exacerbates their overall psychological stress. This persistent stress can significantly elevate the risk of mental health issues, such as depression and anxiety, among sexual minorities [17–19]. Therefore, it is reasonable to propose that cyberbullying may serve as a mediating factor between sexual minority status and mental health outcomes. In this study, we employed propensity score matching to control for confounding factors and, for the first time, utilized causal mediation analysis to investigate the mechanisms linking sexual minority status, cyberbullying, and mental health within the college student population. Our findings aim to illuminate the potential mediating role of cyberbullying in this relationship.

## Methods

### Participants and procedures

The study was conducted in Hubei, a central province in China recognized for its prominent higher education institutions, during the period from October to November 2023. Utilizing convenience sampling, three well-known public universities were selected: Hubei University of Science and Technology, Jingzhou College, and Yangtze University. Participation in the survey was entirely voluntary, and oral informed consent was obtained from all participants prior to data collection. To ensure confidentiality and anonymity, students were assured that their responses would be kept private.

The questionnaire took approximately 10–15 min to complete and was administered either during class meetings or during evening self-study sessions facilitated by the universities. The research protocol received approval from the medical ethics committee at the Ethics Committee of Health Science center, Yangtze University (Number: 202302005).

Out of 2915 students who initially filled out the questionnaires, 23 were excluded due to logical inconsistencies in their answers, yielding a final sample size of 2892 valid surveys and an impressive response rate of 99.2%.

### Measures

#### Basic demographic variables

Key demographic information was gathered, including age, gender, academic year, whether the participant was a single child, and their field of study. In addition, participants were asked to provide self-assessments regarding their subjective socioeconomic status.

#### Sexual minorities

Sexual minorities were assessed using a single item: “Are you part of a sexual minority group (including homosexuals, transgender individuals, men who have sex with men, and other sexual minority populations)?” Participants responded with either “yes” or “no.”

#### Depression anxiety and stress

In this study, we utilized the Chinese version of the Depression Anxiety and Stress Scale-21 (DASS-21) to assess psychological distress among the participants [20]. The DASS-21 comprises three subscales: depression, anxiety, and stress, with each subscale containing seven items. Example items include: “I was unable to become enthusiastic about anything” for the depression subscale, “I felt scared without any good reason” for anxiety, and “I tended to over-react to situations” for stress. Participants rated each item using a four-point Likert scale, reflecting how well the statements described their experiences over the previous week. Each higher total subscale scores indicate greater levels of psychological distress. The DASS-21 has been widely used in studies involving Chinese populations [21, 22]. In this study, the Cronbach’s α = xxx for each subscale. 0.83 for stress, 0.84 for anxiety, 0.83 for depression.

#### Sleep quality

Sleep quality was evaluated using the Single-Item Sleep Quality Scale (SQS) developed by Snyder et al. (2018) [23]. Participants were asked to respond to the question, “How would you rate your overall sleep quality during the past seven days?” They rated their sleep on an 11-point scale, with values ranging from 0 (indicating terrible sleep) to 10 (indicating excellent sleep). A higher score reflects better sleep quality. This scale has been widely used to measure sleep quality in the Chinese population [24, 25].

#### Loneliness

The Three-Item Loneliness Scale was utilized to assess loneliness among participants [26]. This scale employs a 3-point Likert format, allowing responses of “hardly ever,” “some of the time,” or “often.” One item from the scale asks, “How frequently do you feel that you lack companionship?” Total scores are derived by summing the individual item scores, which can range from 3 to 9. Higher total scores indicate a greater degree of loneliness. The Chinese adaptation of the scale has demonstrated strong reliability and validity [27, 28]. In this study, the Cronbach’s alpha for the scale was found to be 0.84.

#### Cyberbullying

The Cyberbullying Scale is a specialized instrument designed to measure cyberbullying behavior, comprising 12 items rated on a 5-point Likert scale (0 = “never” to 4 = “always”) [29]. The items encompass various forms of cyberbullying, such as spreading online rumors and employing offensive or hurtful language. By calculating the total score, researchers can assess the severity of cyberbullying experienced by an individual, with higher scores indicating more severe instances of cyberbullying. This scale has been thoroughly validated in China and is widely utilized in related research [30, 31]. In this study, the Cronbach’s alpha for the scale was found to be 0.92.

### Statistical analysis

#### Descriptive analysis

In this study, qualitative data were expressed in terms of frequencies (N) and percentages (%), while quantitative data were presented as mean ± standard deviation (SD).

#### Propensity score matching (PSM) analysis

To effectively control for potential confounding variables, we employed PSM analysis. The core principle of PSM is to estimate, based on observed baseline characteristics, the probability (propensity score) of each individual receiving a specific treatment or being assigned to a particular group. By carefully matching individuals from the treatment and control groups with similar propensity scores, we created an environment approximating a randomized experiment, significantly reducing bias. In our study, the PSM implementation followed a series of rigorous steps: first, variable selection, where we meticulously screened baseline socio-demographic characteristics that could influence both the outcome variables and treatment assignment; second, propensity score estimation, calculate the conditional probability of belonging to the sexual minority group based on each participant’s baseline characteristics; third, the matching process, where we applied nearest neighbor matching with a caliper of 0.05, ensuring that each participant in the experimental group (sexual minority) was matched with the closest participant in the control group (non-sexual minority college students), with a maximum propensity score difference of 0.05, while maintaining a 1:1 case-control ratio to preserve group balance; and finally, matching quality assessment, where we ensured the effectiveness of the matching by evaluating the balance of baseline characteristics between the matched groups.

#### Correlation analysis and causal mediation analysis

In this study, we utilized Spearman’s correlation analysis to investigate the relationships among our key variables: sexual minorities, cyberbullying, stress, anxiety, depression, sleep quality, and loneliness. Following this, we conducted causal mediation analysis to decompose the total effect of the outcomes into direct and indirect effects, with the indirect effect mediated by specific variables. The analysis report included the average causal mediation effect (ACME), average direct effect (ADE), and total effect. Cyberbullying served as the mediator variable, and we examined its mediating role between sexual minorities and mental health outcomes. A bootstrapping test was employed to calculate the correlation coefficients for the causal mediation effects.

All statistical analyses were performed using SPSS version 21.0, while the causal mediation model was executed in R software version 4.1.3 (mediation package). Significance was established at *P* < 0.05 (two-tailed) for this study.

## Results

This study included a total of 2892 college students as survey participants. Among them, 204 students identified as belonging to sexual minority groups, representing 7.1% of the overall sample. The gender distribution was relatively balanced, with 1499 male students (51.8%) and 1393 female students (48.2%). In terms of academic standing, first-year students comprised the largest proportion at 76.9%, totaling 2224 students. Additionally, 909 students came from one-child families, accounting for 31.4% of the sample. Regarding professional distribution, students majoring in medical fields held the highest proportion at 27.3% (790 students). The average age of the participants was 18.55 years, with a standard deviation of 1.08 years. The average socioeconomic status score was 4.48, accompanied by a standard deviation of 1.62. Detailed demographic data can be found in Table 1.

**Table 1:** The socio-demographic characteristics of participants before and after PSM

Variables	*N* (%)	Before PSM analysis (*N* = 2892)		χ^2^	*P*	After PSM analysis (*N* = 408)		χ^2^	*P*
		Sexual minorities (*N* = 204)	None-sexual minorities (*N* = 2688)			Sexual minorities (*N* = 204)	None-sexual minorities (*N* = 204)		
Sex				**75.39**	**< 0.001**			0.057	0.811
Men	1499(51.8%)	46(22.5%)	1453(54.1%)			46(22.5%)	44(21.6%)		
Women	1393(48.2%)	158(77.5%)	1235(45.9%)			158(77.5%)	160(78.4%)		
Grade				3.60	0.166			0.149	0.928
The first year	2224(76.9%)	146(71,6%)	2078(77.3%)			146(71,6%)	146(71.6%)		
The second year	264(9.1%)	22(10.8%)	242(9.0%)			22(10.8%)	20(9.8%)		
The Junior year or higher	404(14.0%)	36(17.6%)	368(13.7%)			36(17.6%)	38(18.6%)		
Only children				**6.17**	**0.013**			0.260	0.610
Yes	909(31.4%)	80(39.2%)	829(30.8%)			80(39.2%)	75(36.8%)		
No	1983(68.6%)	124(60.8%)	1859(69.2%)			124(60.8%)	129(63.2)		
Academic field				**10.46**	**0.033**			0.116	0.998
Science	344(11.9%)	18(8.8%)	326(12.1%)			18(8.8%)	19(9.3%)		
Engineering	676(23.4%)	38(18.6%)	638(23.7%)			38(18.6%)	40(19.6%)		
Medicine	790(27.3%)	72(35.3%)	718(26.7%)			72(35.3%)	70(34.3%)		
Agriculture	398(13.8%)	23(11.3%)	375(14.0%)			23(11.3%)	23(11.3%)		
Others	684(23.7%)	53(26.0%)	631(23.5%)			53(26.0%)	52(25.5%)		
	Mean ± SD	Mean ± SD	Mean ± SD	T	P	Mean ± SD	Mean ± SD	T	P
Age	18.55 ± 1.08	18.48 ± 1.13	18.56 ± 1.07	1.047	0.306	18.48 ± 1.13	18.49 ± 1.15	0.008	0.931
Subjective socio-economic status	4.48 ± 1.62	4.37 ± 1.69	4.49 ± 1.61	1.032	0.310	4.37 ± 1.69	4.39 ± 1.64	0.022	0.882

### Results of propensity score matching analysis

**Fig. 1:**
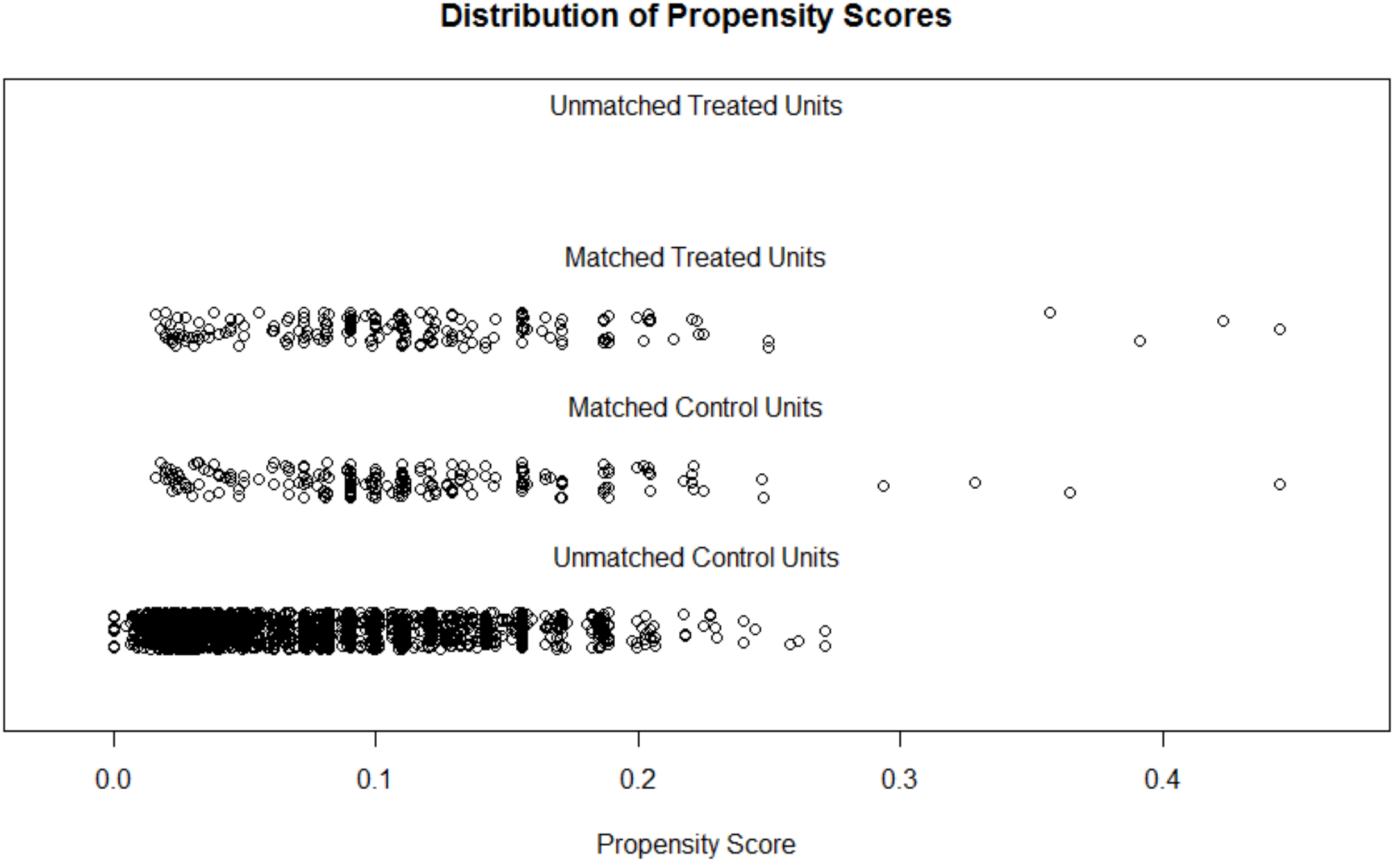
The distribution of propensity score before and after PSM analysis

To assess the impact of confounding variables, this study employed propensity score matching to align samples between sexual minority and non-sexual minority groups. Changes in sample size and propensity scores before and after matching were plotted for both groups (see Fig. 1; Table 1). The standardized mean difference was 0.1758 before matching and had decreased to 0.0015 after matching. To validate the effectiveness of the matching process, tests were conducted on the general demographic characteristics of the sexual minority group prior to and following the matching procedure. For instance, when examining the characteristic of being an only child, the chi-square test result before matching was χ^2^ = 10.46, *p* = 0.013, indicating significant differences between the two groups. Conversely, the chi-square test result after matching was χ^2^ = 0.26, *p* = 0.610, suggesting that various confounding factors were effectively controlled within the sexual minority group. This demonstrates that the matching method succeeded in mitigating the influence of confounding variables on the research findings.

#### Results of correlation analysis

As shown in Table 2, the characteristics of our key variables were as follows: depressive symptoms: 8.94 ± 7.72; anxiety symptoms: 8.12 ± 7.32; stress: 9.13 ± 7.81; sleep quality: 7.62 ± 2.24; cyberbullying: 5.42 ± 6.41; loneliness: 5.05 ± 1.68. Our analysis revealed significant correlations among these key variables, including sexual minority status and psychological factors such as depressive symptoms, anxiety symptoms, stress, cyberbullying, and loneliness. Specifically, being a sexual minority was positively associated with depressive symptoms, anxiety symptoms, stress, cyberbullying, and loneliness, while it was negatively associated with sleep quality.

**Table 2:** The correlation analysis of key characteristics in this study

Variables	Mean ± SD	1	2	3	4	5	6	7
1. Sexual minorities	-	1						
2. Depressive symptoms	8.94 ± 7.72	0.267^**^	1					
3. Anxiety symptoms	8.12 ± 7.32	0.208^**^	0.697^**^	1				
4. Stress	9.13 ± 7.81	0.183^**^	0.704^**^	0.782^**^	1			
5. Sleep quality	7.62 ± 2.24	− 0.098^*^	− 0.400^**^	− 0.400^**^	− 0.436^**^	1		
6. Cyberbullying	5.42 ± 6.41	0.276^**^	0.434^**^	0.494^**^	0.503^**^	− 0.330^**^	1	
7. Loneliness	5.05 ± 1.68	0.219^**^	0.450^**^	0.486^**^	0.562^**^	− 0.327^**^	0.391^**^	1

#### Results of causal mediation analysis

**Fig. 2:**
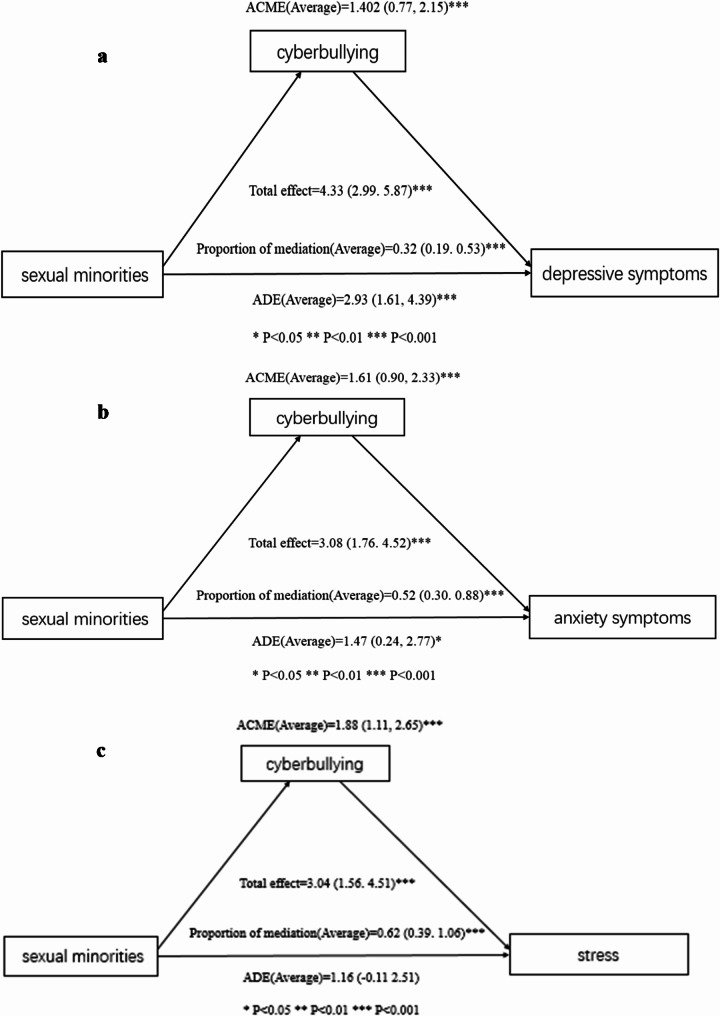

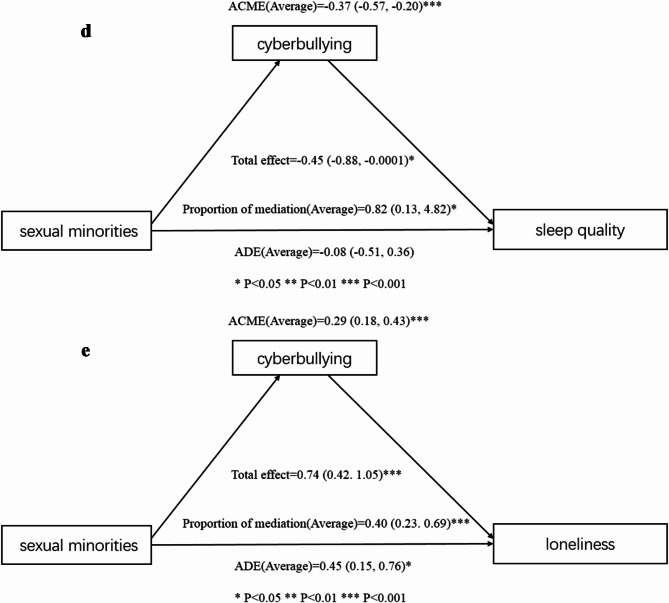
**a** Causal mediation analysis of cyberbullying in the relationship between sexual minorities and depressive symptoms among the participants. **b** Causal mediation analysis of cyberbullying in the relationship between sexual minorities and anxiety symptoms among the participants. **c** Causal mediation analysis of cyberbullying in the relationship between sexual minorities and stress among the participants. **d** Causal mediation analysis of cyberbullying in the relationship between sexual minorities and sleep quality among the participants. **e** Causal mediation analysis of cyberbullying in the relationship between sexual minorities and loneliness among the participants. *ACME* average causal mediation effect, *ADE* average direct effect, *Proportion mediated* = ACME(Average)/total effect

Figure 2 presents the detailed results of the causal mediation analysis.

Figure 2a: The analysis indicated that both direct and indirect effects significantly contributed to the increased odds of depressive symptoms among sexual minorities, with cyberbullying acting as a mediating factor. The total effect was 4.33 (95% CI 2.99–5.87; *P* < 0.001). The Average Causal Mediation Effect (ACME) was 1.402 (95% CI 0.77–2.15; *P* < 0.001), while the Average Direct Effect (ADE) was 2.93 (95% CI 1.61–4.39; *P* < 0.001). The proportion of the effect that was mediated was 0.32 (95% CI 0.19–0.53; *P* < 0.001).

Figure 2b: Similarly, for anxiety symptoms, both direct and indirect effects were found to be significant. The total effect was 3.08 (95% CI 1.76–4.52; *P* < 0.001), with an ACME of 1.61 (95% CI 0.90–2.33; *P* < 0.001) and an ADE of 1.47 (95% CI 0.24–2.77; *P* < 0.05). The proportion of the effect mediated was 0.52 (95% CI 0.30–0.88; *P* < 0.001).

Figure 2c: For stress, the findings highlighted indirect effects. The total effect was 3.04 (95% CI 1.56–4.51; *P* < 0.001), with an ACME of 1.88 (95% CI 1.11–2.65; *P* < 0.001). The ADE was 1.16 (95% CI − 0.11 to 2.51; *P* > 0.05), and the proportion of the effect mediated was 0.62 (95% CI 0.39–1.06; *P* < 0.001).

Figure 2d: In terms of sleep quality, the results showed that indirect effects played significant roles. The total effect was − 0.45 (95% CI − 0.88 to − 0.0001; *P* < 0.05), with an ACME of − 0.37 (95% CI − 0.57 to − 0.20; *P* < 0.001) and an ADE of − 0.08 (95% CI − 0.51 to 0.36; *P* > 0.05). The proportion of the effect mediated was 0.82 (95% CI 0.13–4.82; *P* < 0.05).

Figure 2e: Lastly, regarding loneliness, both direct and indirect effects were again significant. The total effect was 0.74 (95% CI 0.42–1.05; *P* < 0.001), with an ACME of 0.29 (95% CI 0.18–0.43; *P* < 0.001) and an ADE of 0.45 (95% CI 0.15–0.76; *P* < 0.05). The proportion of the effect mediated was 0.40 (95% CI 0.23–0.69; *P* < 0.001).

## Discussion

This study analyzed the association between sexual minorities, cyberbullying, and mental health outcomes. The results indicated that cyberbullying served as a partial mediator between sexual minority status and various mental health issues, specifically depressive symptoms, anxiety symptoms, and loneliness. The proportions of the effects mediated by cyberbullying were 0.32 (95% CI 0.19–0.53; *P* < 0.001) for depressive symptoms, 0.52 (95% CI 0.30–0.88; *P* < 0.001) for anxiety symptoms, and 0.40 (95% CI 0.23–0.69; *P* < 0.001) for loneliness. Furthermore, cyberbullying was found to play a complete mediating role between sexual minorities and both stress and sleep quality. Additionally, we identified 204 students who classified themselves as belonging to sexual minority groups, representing 7.1% of the overall sample. This percentage is lower than the findings from Yun’s study, which reported a higher prevalence. This cross-sectional study utilized data from 54,580 youths collected during the 2019–2020 National College Student Survey on Sexual and Reproductive Health, conducted across 31 provinces in mainland China, where sexual minorities accounted for 22.4% of respondents [32]. In contrast, data from the 2019 School-Based Chinese College Students Health Survey (SCCSHS) showed that only 4.2% of students self-reported as sexual minorities [33]. The discrepancies in prevalence rates may be attributed to the differing purposes and sampling methods employed in each study.

In this comprehensive study, our findings clearly indicate that sexual minorities experience significantly higher rates of cyberbullying compared to their peers. This aligns with previous research, reaffirming the systemic unfair treatment faced by sexual minorities in online spaces [34, 35]. Within the context of traditional Chinese culture, this issue is particularly pronounced. The marginalized status of sexual minorities within established beliefs and social structures subjects them to immense pressure from both society and family in their daily lives [36]. This, coupled with additional challenges and bullying in the online environment, intensifies their vulnerability to cyberbullying and harassment. Consequently, this dual burden exacerbates their overall predicament.

Our findings also reveal that cyberbullying acts as a partial mediator between sexual minority status and various mental health issues, particularly depressive symptoms, anxiety, and feelings of loneliness. This underscores the complex impact of cyberbullying on the mental health of sexual minorities. Minority stress theory provides valuable insights into understanding this [37]. It is not merely an act of aggression; rather, it exacerbates the multifaceted challenges they face on both social and psychological levels. In online spaces, sexual minorities often endure unwarranted abuse, exclusion, and discrimination [38]. These persistent negative experiences further erode their social support networks, intensifying feelings of loneliness and helplessness. Over time, the accumulation of these adverse emotions and psychological pressures can lead to severe mental health problems [39]. Thus, it is crucial for us to acknowledge the significant harm that cyberbullying inflicts on sexual minorities. We must implement more comprehensive and effective prevention and intervention measures to safeguard their mental health and well-being. By doing so, we can help ensure that they are able to express themselves freely and achieve self-realization in a safe and inclusive online environment.

This study highlights the complex interplay between sexual minorities, cyberbullying, and their experiences of stress and sleep quality. The finding that cyberbullying serves as a complete mediator between sexual minority status and both stress and sleep quality suggests that the direct influence of sexual minority status on these outcomes may be more intricate than previously understood. Several key factors contribute to this mediation. Sexual minorities often encounter societal stigma and discrimination, which manifest in online environments through cyberbullying. This hostile atmosphere creates a chronic stressor that exceeds the impact of an individual’s sexual identity alone, explaining why sexual minority status may not directly lead to stress or poor sleep quality without considering the mediating effect of cyberbullying. The online realm provides a platform for anonymous and often unfiltered expressions of prejudice, amplifying the negative effects of discrimination faced by sexual minorities [40]. In this context, cyberbullying becomes a tangible and relentless form of victimization that directly influences stress levels and disrupts sleep patterns, thereby acting as a more immediate cause of these outcomes [41, 42]. Furthermore, the psychological impact of cyberbullying is profound, triggering a cascade of negative emotions and cognitive responses, such as anxiety, fear, and hypervigilance. These emotional states are closely linked to increased stress and disturbances in sleep quality [43, 44]. Consequently, it is this emotional turmoil—rather than sexual minority status itself—that is more likely to directly affect stress levels and sleep quality.

Based on the findings of this study, we propose the following targeted intervention recommendations: Firstly, mental health education for sexual minority groups should be strengthened to enhance their awareness and coping abilities regarding cyberbullying. Secondly, schools should establish professional psychological support services, including counseling and psychotherapy, to assist sexual minority groups in effectively alleviating the psychological pressure caused by cyberbullying. Thirdly, it is necessary to establish and improve anti-cyberbullying mechanisms, and increase supervision and punishment of cyberbullying behaviors. Moreover, the significance of our research extends beyond this, as its findings can also provide important insights for the design of prevention programs. Specifically, we suggest further strengthening collaboration among schools, families, and various sectors of society to jointly construct a comprehensive and multi-layered protective network [45]. Through systematic education and training, public awareness of the harmful effects of cyberbullying should be enhanced, particularly by improving the ability of parents and educators to identify cyberbullying behaviors and intervene effectively. At the same time, sexual minority groups should be encouraged to actively participate in social activities to expand their social support networks [46], enabling them to feel the care and acceptance of society, and thus face various challenges in cyberspace with greater confidence.

## Limitations

Our study has several limitations. Firstly, there is a potential for recall bias social desirability bias and self-reporting bias in the data, as Recall bias stems from inaccurate memories, social desirability bias from responding to please others, and self-reporting bias from intentional or unintentional misreporting. Secondly, this study used convenience sampling in Hubei Province, China, which may lead to a non-representative sample due to cultural and regional differences. We acknowledge potential selection bias as participants may have specific characteristics, like high internet usage or belonging to certain social circles. Therefore, our findings have limitations in generalizability. Future research will consider more scientific sampling methods, such as random or stratified sampling, to enhance sample representativeness and study generalizability. Thirdly, this study used a cross-sectional design with single-point data collection, limiting causal inference. Although we measured cyberbullying using a specialized scale and believe it’s a significant factor in mental health issues, we cannot fully confirm direct causality due to design limitations. Additionally, common underlying causes, such as internet usage habits or lack of social support, cannot be excluded. To accurately explore causal relationships, future research should use a longitudinal design with multi-point data collection to overcome these limitations. Fourthly, despite using PSM to reduce confounding bias, our study faced a limitation of insufficient control of confounding variables. Other critical factors, such as family support, social discrimination, and economic status, were not included in the analysis. Future research will consider these confounding factors more comprehensively and attempt to incorporate them into the analysis. Fifthly, a limitation of this study is the small proportion of sexual minority participants, accounting for only 7.1% (204 individuals) of the sample. This sample size is insufficient for in-depth subgroup analyses. Future research could further explore this area. Sixthly, a limitation of this study is that we used a single scale to measure cyberbullying without further differentiating specific forms of bullying (such as direct attacks, covert harassment, public humiliation) or examining differences across platforms (such as social media, anonymous forums). This may have resulted in an incomplete understanding of the impact of cyberbullying on the mental health of sexual minority individuals. Finally, our study has an important limitation: the exclusion of all potential key social factors as mediating variables. We recognize that, in addition to studied variables, social factors like social support and discrimination may also play significant mediating roles between sexual minority groups and mental health. However, due to study scope and resource constraints, these potential mediating variables were not included. Future research could explore their influence for a more comprehensive understanding.

## Conclusion

This study has explored the complex relationship between sexual minorities, cyberbullying, and mental health outcomes. The results indicate that cyberbullying acts as a partial mediator between sexual minority status and various mental health issues, specifically depressive symptoms, anxiety symptoms, and loneliness. Moreover, cyberbullying fully mediates the relationship between sexual minority status and both stress and sleep quality. These findings underscore the significant impact of cyberbullying on the mental health of sexual minorities, suggesting it is a critical factor to consider when addressing the mental health challenges faced by this population. Therefore, it is essential to develop more comprehensive and effective prevention and intervention measures to protect the mental health and well-being of sexual minorities.

## Data Availability

No datasets were generated or analysed during the current study.

## Abbreviations

DASS-21: The Depression Anxiety and Stress Scale-21
ACME: The average causal mediation effect
ADE: Average direct effect
